# Overview of the *Solar Radiation and Climate Experiment* (SORCE) Seventeen-Year Mission

**DOI:** 10.1007/s11207-021-01869-3

**Published:** 2021-08-23

**Authors:** Thomas N. Woods, Jerald W. Harder, Greg Kopp, Debra McCabe, Gary Rottman, Sean Ryan, Martin Snow

**Affiliations:** 1 Laboratory for Atmospheric and Space Physics, University of Colorado, 3665 Discovery Dr., Boulder, CO 80303 USA; 2 South African National Space Agency, Hospital Street, Hermanus, 7200 South Africa

**Keywords:** Total solar irradiance, Solar spectral irradiance, Sun-climate observations, SORCE mission operations

## Abstract

The *Solar Radiation and Climate Experiment* (SORCE) was a NASA mission that operated from 2003 to 2020 to provide key climate-monitoring measurements of total solar irradiance (TSI) and solar spectral irradiance (SSI). Three important accomplishments of the SORCE mission are i) the continuation of the 42-year-long TSI climate data record, ii) the continuation of the ultraviolet SSI record, and iii) the initiation of the near-ultraviolet, visible, and near-infrared SSI records. All of the SORCE instruments functioned well over the 17-year mission, which far exceeded its five-year prime mission goal. The SORCE spacecraft, having mostly redundant subsystems, was also robust over the mission. The end of the SORCE mission was a planned passivation of the spacecraft following a successful two-year overlap with the NASA *Total and Spectral Solar Irradiance Sensor* (TSIS) mission, which continues the TSI and SSI climate records. There were a couple of instrument anomalies and a few spacecraft anomalies during SORCE’s long mission, but operational changes and updates to flight software enabled SORCE to remain productive to the end of its mission. The most challenging of the anomalies was the degradation of the battery capacity that began to impact operations in 2009 and was the cause for the largest SORCE data gap (August 2013 – February 2014). An overview of the SORCE mission is provided with a couple of science highlights and a discussion of flight anomalies that impacted the solar observations. Companion articles about the SORCE instruments and their final science data-processing algorithms provide additional details about the instrument measurements over the duration of the mission.

## Science Overview

The *Solar Radiation and Climate Experiment* (SORCE) provided solar-irradiance observations during its 17 years of operations. These measurements are both crucial and unique for achieving NASA’s goals in support of US and international policy on climate change. Solar irradiance is the principal energy input to the global climate system and is critical for studying the radiative-energy balance, atmosphere photochemistry (such as effects on ozone), and solar influence on global and regional climate change (e.g. Lean et al., 2005; Pilewskie et al., 2005; Ermolli et al., 2013). Collecting accurate solar-irradiance data spanning multiple years is crucial for understanding how much solar radiation is deposited in the Earth’s atmosphere and at the surface, and thus how much energy is available to influence weather, climate, the cryosphere, atmosphere dynamics, and ocean currents. Because of its relevance for natural climate forcings at any given time, the total solar irradiance (TSI) is identified within the President’s National Plan for Civil Earth Observations (Holdren, 2014) as a vital observation for determining the Earth’s net energy balance. Similarly, within NASA’s Science Plan (2014) and NOAA’s Climate Data Record (CDR) program, both the TSI and the solar spectral irradiance (SSI) between 200 nm and 2400 nm are recognized as important long-term measurements for a robust, sustainable, and scientifically defensible approach in climate-change research. The first NOAA CDRs for TSI and SSI were released in 2016 (Coddington et al., 2016) and are based on SORCE observations. The Inter-governmental Panel on Climate Change (IPCC) report (2013) also recognizes the importance of the ongoing solar-irradiance measurements and the need to further improve the accuracy of the solar radiative forcing on climate through heating the surface directly with visible and near-infrared radiation and through heating the atmosphere with solar ultraviolet radiation.

SORCE’s *Total Irradiance Monitor* (TIM: Kopp and Lawrence, 2005; Kopp, Heuerman, and Lawrence, 2005) is an ambient-temperature electrical substitution radiometer (ESR) with four channels, with one channel used for the daily TSI observations and other channels used less frequently to track instrument degradation trends. SORCE/TIM measures the TSI with accuracy and repeatability capable of resolving how solar irradiance varies and how these variations affect climate. Kopp (2021) discusses these TSI measurements over the duration of the SORCE mission. The *Spectral Irradiance Monitor* (SIM: Harder et al., 2005a,b) (240 – 2413 nm), *SOLar STellar Irradiance Comparison Experiment* (SOLSTICE: McClintock, Rottman, and Woods, 2005; McClintock, Snow, and Woods, 2005) (115 – 308 nm), and *X-ray UV Photometer System* (XPS: Woods and Rottman, 2005; Woods, Rottman, and Vest, 2005) (0.1 – 40 nm and H i 121.6 nm) measure the spectral composition of the total irradiance. The SORCE/SIM is a dual-channel prism spectrometer that measures the SSI in the near-ultraviolet, visible, and near-infrared ranges. One SIM channel is used for the daily SSI observations, and the other channel is used about once a month to track instrument degradation. The SORCE/SOLSTICE is a set of two grating spectrometers for measuring the SSI in the far-ultraviolet and middle-ultraviolet ranges. Both SOLSTICE-A and SOLSTICE-B are used daily for solar observations, and stellar observations of stable main-sequence O–B stars are used to track the degradation of the SOLSTICE channels. The SORCE/XPS is a set of photometers with bandpass filters covering the extreme-ultraviolet and soft X-ray ranges. The SORCE/SIM, SORCE/SOLSTICE, and SORCE/XPS observations and their latest data-processing algorithms are discussed in more detail by Harder et al. (2021), Snow et al. (2021), and Woods and Elliott (2021), respectively. These SSI measurements and their variabilities used in climate models facilitate the understanding of climate-change. In particular, climate models used for the *IPCC Fifth Assessment Report* (2013) include radiative and photochemical schemes sufficiently advanced to utilize SSI as input, not just the TSI that was the standard input in climate-model simulations used for the earlier IPCC reports. The *Solar Physics* Topical Issue about SORCE (2005) provides details about the designs and calibrations of the SORCE instruments, as well as some early science results. A summary of several science highlights from SORCE is provided by Woods et al. (2013) after SORCE had been in orbit for ten years.

**Figure 1:**
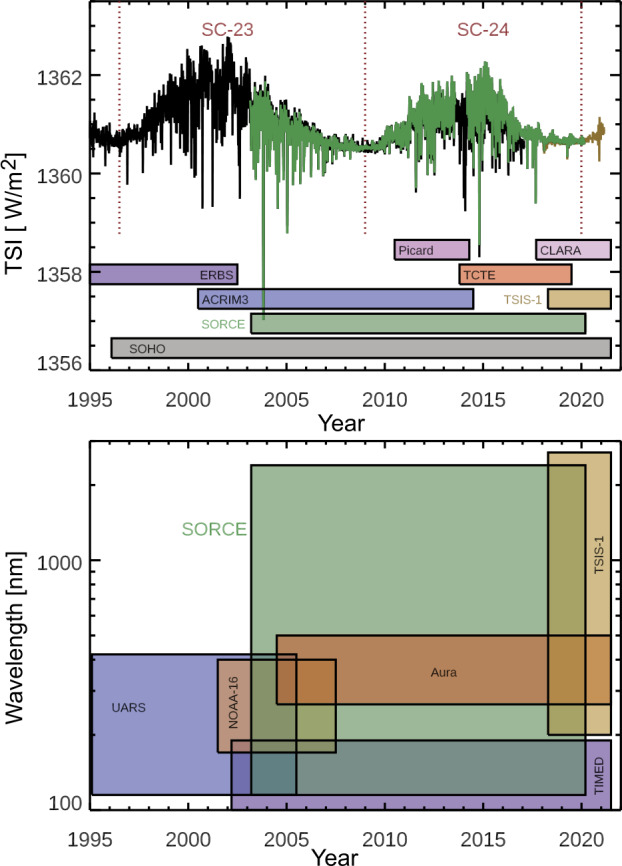
The SORCE solar observations started in February 2003 during the declining phase of Solar Cycle 23, continued over Solar Cycle 24, and ended in February 2020 at the minimum transition into Solar Cycle 25. The TSI variations from SORCE (green), SOHO (black), and TSIS-1 (gold) are shown over this period in the top panel. The SOHO and TSIS-1 TSI time series are adjusted slightly to the SORCE TSI level. The missions with SSI daily observations are indicated in the bottom panel.

**Figure 2:**
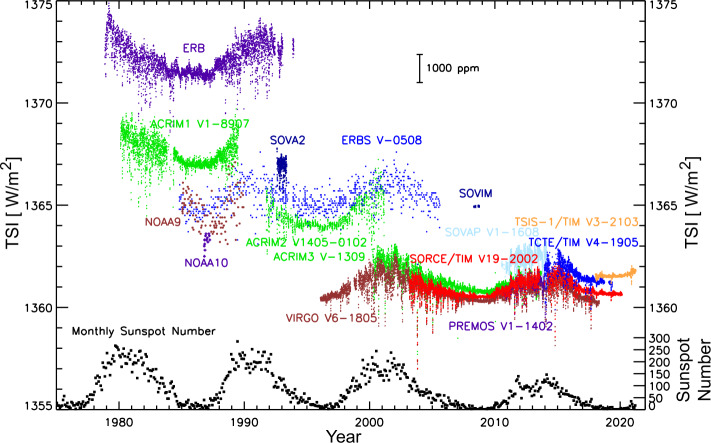
The spacecraft TSI climate record started in 1978 during Solar Cycle 21. The SORCE/TIM TSI observations are over 17 years of this 42-year TSI record. The SORCE TIM (red) established the lower TSI value that now has consensus in the TSI community.

**Figure 3:**
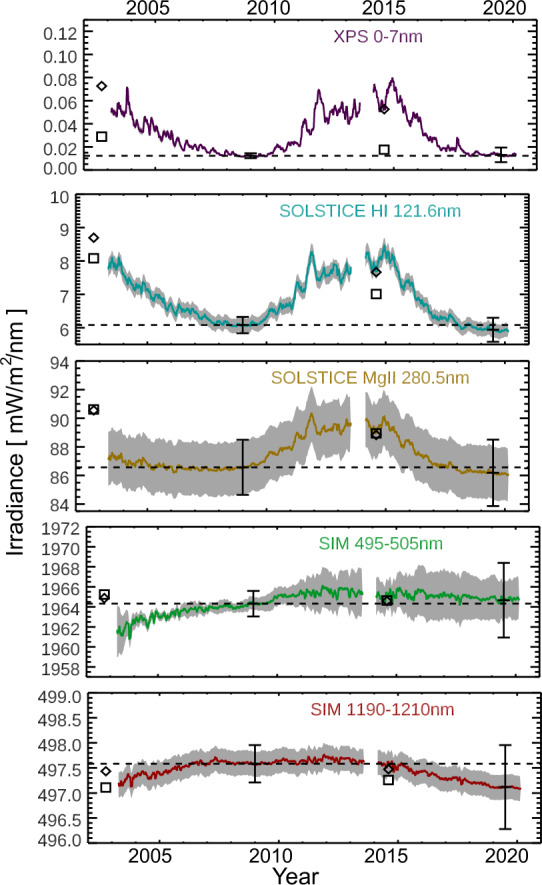
The SORCE SSI observations span 17 years, covering the declining phase of Solar Cycle 23, cycle minimum in 2008 – 2009, all of Solar Cycle 24, and the next cycle minimum in 2019. The select wavelengths are examples for emissions from the photosphere (500 nm and 1200 nm), chromosphere (280.5 nm), transition region (121.6 nm), and corona (0.1 – 7 nm). The variability for the visible and NIR wavelengths is dominated by dark sunspots and bright faculae, and the UV variability has only bright plage contributions from active regions and active network. The gray regions bound the irradiance with its one-sigma measurement uncertainty. The error bars at cycle minima (2009 and 2019) include the measurement uncertainties and the stability estimates from the beginning of the mission (2003). These times series have 27-day smoothing of the SORCE Level 3 data. The Solar Cycle 23 maximum value in 2002 and Solar Cycle 24 maximum value in 2014 are shown for the SSI3 composite (diamonds) and Solar Irradiance Data (SOLID) composite (squares) with normalization relative to the SORCE solar-cycle minimum value in 2009.

A key motivation for extending the SORCE measurements well past its five-year prime mission was to prevent a gap in the TSI and SSI climate records, as shown in Figures 1 – 3 and discussed more in Sections 2 and 3. With the launch of TSIS-1 to the *International Space Station* (ISS) in December 2017 and TSIS-1 routine solar observations beginning in March 2018, there was a 22-month overlap of SORCE and TSIS-1 observations. Because pre-flight calibration uncertainties are typically ten times greater than the measurement precision of comparing overlapping measurements, maintaining the quality of TSI and SSI records relies on having sufficient overlapping data sets. For example, the SORCE/TIM relative accuracy was about 350 ppm at launch, but its measurement precision is better than 10 ppm. Therefore, using the overlapping measurements of SORCE and TSIS-1 one can establish a TSI record at the time of their overlap with a relative uncertainty of about 20 ppm. In contrast, if there were no overlap, the TSI record would have instead a 500-ppm uncertainty for the transition.

SORCE’s solar-irradiance observations have advanced our understanding of solar radiative forcing of Earth’s climate and atmosphere during the descending phase of Solar Cycle 23 for SORCE’s prime mission in 2003 – 2008. SORCE’s extended mission has continued its TSI and SSI records through an unusually extended minimum at the end of Cycle 23 in 2008 – 2009 and over the full period of Solar Cycle 24 (2009 – 2020), which is the least active cycle in 90 years. The SORCE data set is also remarkable in observing two solar-cycle minima periods with the same instruments, so estimates of the solar-irradiance secular trends are possible (Woods et al., 2021). The following provides a few examples of Sun–climate studies with SORCE data. Furthermore, Ermolli et al. (2013) and the references therein provide more detailed research results.

L’Ecuyer et al. (2015) provide a detailed analysis of the radiative energy balance using the most accurate top of the atmosphere (TOA) solar-radiation measurements from SORCE and many radiation measurements from several other NASA spacecraft. These NASA spacecraft measurements, including SORCE, are critically important to constrain the complicated energy flows within the climate system, to resolve disputed energy estimates (Stephens et al., 2012; Wild et al., 2015; L’Ecuyer et al., 2015), and to improve the Clouds and the Earth’s Radiant Energy System (CERES) energy balance and filled data product (Loeb and NCAR Staff, 2018).

Matthes et al. (2016) highlight the importance of the SSI data in simulating the current climate state and ozone photochemistry for state-of-the-art chemistry–climate models. Variations in SSI from Phase 5 to Phase 6 of the Coupled Model Inter-comparison Project (CMIP5 to CMIP6) have led to a −1.5 K cooler upper stratosphere and lower (−3%) ozone abundance in the lower stratosphere. The response of ozone concentration to the 11-year solar cycle depends strongly on how each wavelength of the SSI varies (Swartz et al., 2012). Significant uncertainty and controversy remain because of the short data records used thus far in model simulations (e.g. Haigh et al., 2010; Garcia, 2010). The long SORCE mission spanning two solar cycles has helped to better constrain the solar-cycle variability, so there is good potential that additional modeling with the full SORCE solar-irradiance record could resolve some of this controversy.

Solar variations act in conjunction with volcanic activity, the El Niño Southern Oscillation (ENSO), and anthropogenic gases to alter climate (e.g. Mann et al., 2005; Lean and Rind, 2008). Solar-related changes in global surface temperature have been of order 0.1 °C during the recent solar cycles and are superimposed on a longer-term trend driven by increasing greenhouse-gas concentration. Global warming over the past century is attributable mainly to increasing anthropogenic gases, with solar-irradiance variability estimated to cause about 10% of the 0.74 °C per century increase in global surface temperature (Lean and Rind, 2008). The 17-year observations from SORCE help to better constrain the secular trends of solar variability for long-term climate change studies.

On the shorter timescales of years to decades, changes in solar irradiance modulate the overall century-scale warming, and this natural variability, together with ENSO and volcanic aerosol-driven changes, must be properly specified. Furthermore, accurate characterization of the SSI is necessary for understanding the coupling processes and deposition of solar energy in Earth’s atmosphere and surface, and subsequent effects on ocean and atmosphere dynamics that largely influence regional climate changes. Offsets of anthropogenic warming by decreasing solar irradiance from 2002 to 2009 and ENSO variations are contributing factors for the lack of more significant global surface temperature increase during the previous decade (Kopp and Lean, 2011). Solar Cycle 24 has proven to be the weakest cycle during the past 90 years and Cycle 25 may also be a weak cycle. It will be interesting to see how much this lower solar activity influences global temperatures according to observational and modeling studies. Taking these natural changes into account is expected to improve decadal climate projections and validation (e.g. Meehl, Teng, and Arblaster, 2014).

The TSI and SSI climate records are fundamental for these and other Sun–climate studies. As discussed next, these solar-irradiance climate records span about four decades, and SORCE data are key contributions to them for almost two decades. Notably, three important accomplishments of the SORCE mission are i) the continuation of the 42-year-long TSI climate record, ii) the continuation of the ultraviolet (UV) SSI record, and iii) the initiation of the near-ultraviolet (NUV), visible (Vis), and near-infrared (NIR) SSI records. Following brief discussions of those climate records, the SORCE mission operations are described to provide the context on why there are some data gaps in the SORCE data products. Associated with this article are companion articles about the SORCE instruments and their final science data-processing algorithms (TIM: Kopp, 2021; SIM: Harder et al., 2021; SOLSTICE: Snow et al., 2021; XPS: Woods and Elliott, 2021) and also an additional article about solar-cycle variability (Woods et al., 2021).

## TSI Climate Data Record

The TSI climate data record is not a single record but encompasses several different TSI instrument data sets, which, when combined, provide TSI composite time series. There are many different approaches to combining these individual data sets, each at their own irradiance level and with different precision and stability. Consequently, there are a few different TSI composite time series. The early versions of TSI composites tended to start with a reference data set and to combine the other ones to match that reference TSI level. For example, Fröhlich (2009) used the TSI record from the *Solar and Heliospheric Observatory* (SOHO) *Variability of Solar Irradiance and Gravity Oscillations* (VIRGO: Fröhlich et al., 1995, 1997) and instrument-degradation corrections for trending to establish a reference TSI level. Then he adjusted the earlier *Earth Radiation Budget Satellite* (ERBS) and *Active Cavity Radiometer Irradiance Monitor* (ACRIM) data sets to match the VIRGO level. More recently, an International Space Studies Institute (ISSI) team discussed and then developed the approach of weighting the different TSI data sets by their individual uncertainties to make a new TSI composite using the methodology published by Dudok de Wit et al. (2017). At the time of that writing, the SORCE/TIM and the *Picard PREcision MOnitor Sensor* (PREMOS) instrument (Schmutz et al., 2013) TSI data sets provided the best absolute accuracies of ≈350 ppm, and so their example TSI composite is close to the SORCE/TSI level. Notably, the SORCE/TIM and PREMOS measurements of TSI are about 0.35% lower than the earlier TSI measurements (Kopp and Lean, 2011; Ball et al., 2016; Kopp, 2021). Another approach taken for the NOAA Climate Data Record (CDR) is to use proxy modeling with the SORCE solar-irradiance observations to extend the TSI CDR back to 1882 (Coddington et al., 2016). This proxy model, called the NRLTSI-2, uses a proxy to represent the dark sunspots and another proxy to represent the bright-faculae contributions. While there are multiple TSI composites, and more will be developed as previous data sets are refined and as newer data sets become available, it is clear that the 17-year TSI record from SORCE is an important backbone for the TSI climate record.

While the different TSI composites have similar results for the 27-day solar rotation and 11-year solar-cycle variability, there are differences in the magnitude of the TSI and for the long-term (secular) trends that are best seen when comparing the TSI at different solar-cycle minima. Those long-term trends between cycle minima are typically less than the modern-day TSI accuracy of 300 ppm, so it is not clear that any one TSI composite is superior for determining the true long-term TSI trend. While the TSI trends between cycle minima are small for the spacecraft era (1970s to present), irradiance modeling and reconstructions to past centuries, in particular to the Maunder Minimum solar-quiet period in the 1600s – 1700s, indicate larger changes in the TSI (e.g. Wang, Lean, and Sheeley, 2005; Shapiro et al., 2011; Wu et al., 2018). Consequently, there are ongoing discussions on how low the solar irradiance could have been in the past and the inter-related debates on the relative importance of solar variability and volcanic aerosol forcings causing global cooling during ice ages (e.g. Mann et al., 2005). We do not attempt to address those differences here, but we mention them as examples of why it is important to continue making the most accurate solar-irradiance measurements and to combine them into TSI composites that are as accurate as possible.

The rest of the discussion in this section focuses on the SORCE/TIM TSI observations and their overlap with other TSI observations, as illustrated in Figure 2 and listed in Table 1. The SORCE/ TIM began acquiring TSI measurements in 2003 with an unprecedented accuracy of 350 ppm and stability of 10 ppm year^−1^. The early phase of the SORCE mission provided direct measurement overlap with TSI instruments on the ACRIMSat, ERBS, and SOHO/VIRGO, the first two of which have ceased operations but help provide connection to the prior TSI record. The SORCE/TIM has since acquired direct overlap with the *Picard*/PREMOS instrument and the TSI *Calibration Transfer Experiment* (TCTE: Kopp, 2014) onboard the *Air Force Space Test Program Satellite-3*. Both PREMOS and TCTE observations ceased before the SORCE mission ended. The final phase of the SORCE mission overlaps with the *Total and Spectral Solar Irradiance Sensor*-1 (TSIS-1) TIM and the *NorSat-1*/*Compact Lightweight Absolute Radiometer* (CLARA: Finsterle et al., 2014). For NASA, the TSI record is being continued with TSIS-1/TIM onboard the *International Space Station* (ISS), and TSIS-2 is being developed as free-flyer small spacecraft that could launch in 2024. These later TSI instruments all benefit from calibrations or validations at LASP’s *TSI Radiometric Facility* (TRF: Kopp et al., 2007), which has been helpful for justifying the absolute accuracy of the TSI measurements to about 300 ppm.

**Table 1:** Solar irradiance observations during the SORCE mission.

Spacecraft/Instrument	TSI or SSI wavelength range	SSI spectral resolution	Time range	Instrument key reference
ERBS	TSI	N/A	1984 – 2002	Lee, Barkstrom, and Cess (1987)
**SOHO/VIRGO**	TSI	N/A	1996 – cont.	Fröhlich et al. (1995)
ACRIMSat/ACRIM3	TSI	N/A	2000 – 2014	Willson and Helizon (1999)
*Picard*/PREMOS	TSI	N/A	2010 – 2014	Schmutz et al. (2013)
*Picard*/SOVAP	TSI	N/A	2010 – 2014	Meftah et al. (2016)
TCTE/TIM	TSI	N/A	2013 – 2019	Kopp (2014)
**TSIS-1/TIM**	TSI	N/A	2018 – cont.	Kopp (2014)
**NorSat-1/CLARA**	TSI	N/A	2017 – cont.	Finsterle et al. (2014)
UARS/SOLSTICE	115 – 410 nm	1 nm	1991 – 2005	Rottman, Woods, and Sparn (1993)
UARS/SUSIM	120 – 420 nm	0.1 nm	1991 – 2005	Brueckner et al. (1993)
NOAA-16 SBUV2	170 – 400 nm	1.1 nm	2001 – 2007	DeLand et al. (2019)
**TIMED/SEE**	0.5 – 190 nm	0.4 – 1 nm	2002 – cont.	Woods et al. (2005)
**Aura/OMI**	265 – 500 nm	0.5 – nm	2004 – cont.	Marchenko et al. (2019)
ISS/SOLSPEC	160 – 3800 nm	1.2 – 8 nm	2008 – 2017	Thuillier et al. (2009)
**TSIS-1/SIM**	500 – 1600 nm	1 – 10 nm	2018 – cont.	Richard et al. (2020)

The other TSI and SSI observations during the SORCE mission are listed with their key reference. Missions listed in bold font are the ones that have continued observations past the SORCE mission.

The TRF instrument calibrations helped to validate the SORCE/TIM TSI-values that were 4.5 W m^−2^ lower than those reported by all other on-orbit instruments when SORCE was launched. These lower values were first reported by Kopp, Lawrence, and Rottman (2005) and credibly established by Kopp and Lean (2011). The subsequent PREMOS TSI instrument further substantiated the accuracy of this lower TSI value (Fehlmann et al., 2012; Schmutz et al., 2013). Kopp and Lean (2011) explain that the cause of the values from other instruments being erroneously high is largely due to internal instrument scattered light. Since then, the ACRIM-3 data have been lowered (Willson, 2014) and the newer PREMOS, TCTE, and TSIS measurements helped validate this lower and more accurate TSI-value, the former by transferring calibrations from the ground-based TRF to orbit (Schmutz et al., 2013). The VIRGO values (ftp://ftp.pmodwrc.ch/pub) were adjusted in November 2014 to match those of the SORCE/TIM. In 2016, the SORCE and TCTE programs helped support ground calibrations of engineering units representing the ACRIM-1 and ACRIM-2 at the TRF.

The SORCE/TIM TSI observations have been crucial for the TSI climate data record by establishing a lower TSI level with improved accuracy and by reducing the uncertainties for long-term trends with its remarkable stability of 10 ppm year^−1^. These improvements are starting to be incorporated into many climate studies, as discussed in the previous section. For example, L’Ecuyer et al. (2015) use SORCE’s lower TSI-value in their estimates of global radiative energy balance and thus adjust outgoing short- and long-wave energy values correspondingly. The Coupled Model Inter-comparison Project (CMIP6) now normalizes the SSI record it produces for climate-model inter-comparisons to the lower TSI-value established by SORCE, decreasing the Earth-incident radiant-energy levels from the previous CMIP5-values (Matthes et al., 2016).

## SSI Climate Data Record

The SSI climate data record is far more complex than the TSI climate record due to the several thousand different wavelengths and many gaps both in time and wavelength. The SORCE/SSI measurements have significantly improved the SSI record by providing a 17-year data set and coverage over a wide spectral range. The SORCE/SSI instruments include the SIM for the 240 – 2413 nm range, SOLSTICE for the 115 – 308 nm range, and XPS for the 0.1 – 40 nm range and for the H i 121.6 nm emission. The SORCE/SSI observations have continued the ultraviolet (UV) SSI record that started in 1978 for the 200 – 400 nm range with NOAA’s *Solar Backscatter Ultraviolet* (SBUV: Heath and Schlesinger, 1986) and the record for the 115 – 300 nm range that was started in 1981 by the *Solar Mesospheric Explorer* (SME: Rottman et al., 1982). The complementary *Thermosphere, Ionosphere, Energetics, and Dynamics* (TIMED) spacecraft record in the 0.1 – 195 nm range started in 2002 (Woods et al., 2005). SORCE/SIM is the first to provide daily, calibrated measurements for the SSI record in the 400 – 2400 nm range. There are also complementary SSI observations from the *SCanning Imaging Absorption spectroMeter for Atmospheric CHartographY* (SCIAMACHY) onboard the *Environmental Satellite* (ENVISAT: 2002 – 2012). The SCIAMACHY observations in the 240 – 2380 nm range compare well with SORCE, but degradation trending for SCIAMACHY is more challenging (Pagaran et al., 2011). SORCE also overlapped with the *Upper Atmosphere Research Satellite* (UARS) SSI measurements in the 115 – 400 nm range until the UARS solar measurements ended in August 2005. Figure 3 shows a few example time series for the SSI record during the SORCE mission. The TSIS-1/SIM observations are continuing the 200 – 2400 nm measurements, which span the spectral range most critical for climate studies.

The SSI climate data record includes some solar reference spectra, a few SSI composite records, and some SSI models for estimating the SSI variability in order to fill spectral and temporal gaps and for reconstructions prior to the spacecraft era. For example, Harder et al. (2010) provides a reference NUV-Vis-NIR spectrum from SORCE/SIM and a reference spectrum is provided by the SOLSPEC team using SORCE as validation (Thuillier et al., 2014; Meftah et al., 2018). The SORCE team also provided the 2008 solar-cycle-minimum reference spectra for the Whole Heliosphere Interval (WHI) international campaign (Woods et al., 2009). The more recent SSI composites include the Solar Irradiance Data (SOLID) composite by Haberreiter et al. (2017) for the 0.5 – 1991.5 nm range, the GSFCSSI2 version by DeLand et al. (2019) for the 120 – 400 nm range, and the SSI3 composite by Woods and DeLand (2021) for the 0.5 – 1600 nm range. The NOAA CDR for SSI is similar to the TSI CDR in being a proxy model, called the NRLSSI-2 model, which was developed with the SORCE solar-irradiance observations (Coddington et al., 2016). Lean et al. (2020) have the updated model version called the NRLSSI-3. Another SSI model used for climate studies is the *Spectral And Total Irradiance Reconstruction* (SATIRE) model (Krivova, Solanki, and Unruh, 2011).

There are significant differences in solar-cycle variability between the observed and modeled spectra. As detailed by Ermolli et al. (2013), this spectrally dependent variability is critical for understanding the solar forcings and their impacts in Earth’s climate system at inter-annual and decadal scales. To assist in addressing those concerns, models of the SSI variability are being refined based on the SORCE observations. Ermolli et al. (2013) provide a comparison of model estimates of solar-cycle variability for the NUV-Vis-NIR and show significant differences among the model variability estimates and the early SORCE/SIM results about SSI variability as presented by Harder et al. (2009). The Ermolli et al. (2013) primary concern is that the large NUV in-phase variability and large visible out-of-phase variability from Harder et al. (2009) are not reproduced in the NRLSSI model (Lean et al., 2005) and SATIRE model (Krivova, Solanki, and Unruh, 2011) but are simulated in the Solar Radiation Physical Model (SRPM: Fontenla et al., 2011).

Wehrli, Schmutz, and Shapiro (2013) and Haberreiter et al. (2017) also point out that there are inconsistencies in the SORCE/SIM variability between Solar Cycles 23 and 24, notably at 500 nm. As shown in Figure 3, the 500 nm and 1200 nm irradiance time series both show a rise of irradiance during Solar Cycle 23 decline (2003 – 2009) and a slight irradiance increase during the Solar Cycle 24 rise (2009 – 2013). It seems unlikely that the irradiance at a single wavelength would behave differently during two different solar cycles, but we note that those trends are within their measurement uncertainties that are shown in Figure 3 as the gray regions. Namely, the trends for the 500 nm and 1200 nm irradiance are within the ≈0.1% stability estimate, although more so for the 1200 nm irradiance than for the 500 nm irradiance. The SORCE/SIM final processing algorithms, including more information about the SIM measurement uncertainties and stability estimates, are provided by Harder et al. (2021).

Different approaches have been taken to use the SORCE SSI data for modeling the solar- cycle variations. For example, Haberreiter et al. (2017) use the SORCE/SIM data only from 2010 to 2015 in their SSI composite called SOLID. Mauceri et al. (2020) and Woods et al. (2018) studied the SIM trends and suggest slightly revised trends for the SIM data based on comparing trends to TSI and solar proxies, respectively. The Woods et al. (2018) trending results for SIM are used to make a new SSI composite called SSI3 (Woods and DeLand, 2021), and the solar-cycle variability from this SSI3 composite agrees reasonably well with the Haberreiter et al. (2017) SOLID composite that uses a different approach. As indicated in Figure 3, the composites variabilities for 500 nm have very small increases relative to solar 2009 minimum by 0.03% and 0.015% for Solar Cycles 23 and 24 maxima, respectively. Whereas, the composites variabilities for 1200 nm relative to the 2009 solar minimum have small decreases by −0.07% and −0.03% for Solar Cycles 23 and 24 maxima, respectively. Woods et al. (2021) provide more discussion about the solar-cycle variability results for the SORCE data for these two solar cycles and more in-depth comparisons to other measurements and models. Recognizing some of the challenges of the SORCE/SIM trend analysis with just the two channels on that instrument, the TSIS-1/SIM instrument was improved to have three channels as well as having other improvements from lessons learned with the SORCE/SIM observations. We anticipate further insights to these Solar Cycle 23 and 24 SORCE/SIM measurements from improvements to the solar-cycle dependence of the SSI record as the TSIS-1 mission makes new observations during Solar Cycle 25.

Since the Ermolli et al. (2013) comparisons, Ball et al. (2014) have updated the SATIRE model, and Coddington et al. (2016) and Lean et al. (2020) have updated the NRLSSI model. Additionally, the degradation trends for the SORCE instruments have been refined, bringing measurements and models into better agreement. While the differences between observations and models have decreased, notably in the NUV and visible regions, there remain differences in the phasing of the solar-cycle variability in the NIR region (Woods and DeLand, 2021). The new SSI measurements with improved accuracy and stability by TSIS-1/SIM are anticipated to resolve some of the remaining differences. However, the low solar activity so far during the TSIS-1 mission (2018 to present) does not provide enough solar-cycle variability to accurately address those differences yet. Consequently, the SSI variability in some wavelengths remains controversial and unresolved.

## Mission Operations

The SORCE mission started with its launch on 25 January 2003 into a near-circular, 610-km altitude orbit inclined at 40°. After a two-month commissioning phase, SORCE began normal operations in March 2003 with the goal of obtaining at least four TSI observations per day and at least two SSI observations per day. SORCE far exceeded its five-year prime mission objective and had an extended mission from 2008 to 2020. In not having a propulsion system, the spacecraft altitude dropped about one km per year due to atmospheric drag. The current orbit decay estimates predict that the spacecraft will re-enter (burn up) in Earth’s lower atmosphere in the 2031 timeframe. Due to the limitations of funding and battery-capacity issues, SORCE was passivated (turned off) on 25 February 2020. As planned by NASA, the TSI and SSI observations are being continued by the TSIS-1 mission. The following describes the SORCE mission operations and anomalies that impacted the solar observations (e.g. occasional data gaps).

The SORCE mission operations and science data processing were done at LASP’s Mission Operations Center (MOC) in Boulder, CO with support from NASA’s Goddard Space Flight Center (GSFC in Greenbelt, MD) and Orbital Science Corp. (OSC in Dulles, VA, now Northrop Grumman Innovation Systems). Standard NASA services were used to connect the SORCE MOC to the primary ground stations, which are located at the Wallops Flight Facility in Virginia and at Santiago, Chile. In addition, SORCE communicated via the *Tracking and Data Relay System* (TDRS), which was important for early orbit operations and later in the mission when real-time communications were required every day for science data capture.

The SORCE satellite bus from Orbital was a robust spacecraft designed with significant redundancy. The only non-redundant component on the bus was the battery. The SORCE spacecraft operated through its five-year prime mission before any significant anomalies arose. During the extended mission, the more significant anomalies include a reaction-wheel anomaly identified in 2008, degradation of the battery capacity starting in 2009, and the loss of a star tracker in 2012. The exceptional mission operations team overseeing SORCE addressed each of these issues, allowing SORCE to continue making high-quality TSI and SSI daily observations. The solar-irradiance uncertainties increased somewhat due to the larger temperature variations that the instruments experienced once power cycling around orbit eclipse began in 2009. Anomalies often resulted in a gap of a few days while they were resolved through changing the observation sequences, subsystem re-configuration, or the flight or ground software. Table 2 lists the timeline of the mission anomalies that produced data gaps. The largest gap is a seven-month data gap after the battery anomaly in July 2013 and then full recovery into its new Daylight Only Operations (DO-Op) mode in March 2014. The SORCE instruments and majority of the spacecraft subsystems were power-cycled each orbit in the DO-Op mode. With significant changes made to the flight software in 2013 – 2015, the SORCE operations in this DO-Op mode became very routine. Furthermore, the quality of the solar-irradiance results from SORCE in the DO-Op mode remained high, but there are some increased uncertainties for the TSI and SSI observations due to wider temperature swings of the instruments in the DO-Op mode. As planned by NASA, the SORCE observations were continued until February 2020 in order to have sufficient overlap with the TSIS-1 mission. Each of the significant anomalies are described next.

**Table 2:** SORCE mission and instrument events causing data gaps.

Start date (YYYY/DOY)	Gap (days)	Instruments	Event
2003/056	48	SIM, SOL	First Day of Observations for TIM and XPS. Additional Outgassing Period for SIM and SOLSTICE
2003/057	1	TIM	Contingency mode due to failed star tracker self-test
2003/059	5	TIM	Spontaneous MU reset due to read zero error
2003/065	1	TIM	MU reset
2003/109	1	ALL	Load shed level 2: instruments off
2003/125	3	SIM	SIM prism temperature is out of limit
2003/188	1	TIM	TIM put into safe mode due to orbit ram violations
2005/348	2	XPS	XPS filter wheel anomaly
2007/135	6	ALL	Safehold due to OBC reset
2009/004	11	ALL	Safehold due to OBC reset
2009/288	2	ALL	Contingency mode due to under voltage
2010/270	6	ALL	Safehold due to bad MU packets
2010/305	3	ALL	Safehold due to bad MU packets
2010/308	4	SOL, XPS	Extra days to recover from safehold
2010/361	5	ALL	Safehold due to bad MU packets
2011/001	4	SOL, XPS	Extra days to recover from safehold
2011/029	2	ALL	Contingency mode due to ACS fault
2011/137	2	ALL	MU reset and recovery
2011/250	8	ALL	Contingency mode for swapping APE A to APE B
2012/305	9	ALL	Under voltage level 4 resulted in TIM power cycling, then manually commanded off MU
2012/314	13	ALL	Contingency mode for swapping back to APE A
2012/338	4	SOL	SOLSTICE temperature is out of limit
2012/354	9	TIM	Missing TIM thermistor data
2013/004	6	TIM	Missing TIM thermistor data
2013/197	7	ALL	Contingency mode / safehold due to erroneous transmitter commands and OBC reboot
2013/204	8	SOL	SOLSTICE power was off
2013/212	144	ALL	Spacecraft maintenance only while flight operations team developed and implemented daylight only operations.
2013/363	66	ALL	Terminated science operations due to power concerns. Star tracker turned off in eclipse now
2017/225	1	TIM	Uplink card brown-out
2017/228	1	TIM	Possibly an RTS 17 starting late so observatory returned to safehold. This was not unique to this day, however.
2018/189	1	TIM	TIM temperature is out of limit
2018/245	1	TIM	First contact later in orbit day than nominal resulted in an APE reset then abnormal performance after it booted.
2019/088	1	ALL	APE bad boot and no spacecraft telemetry for 8 orbits
2019/112	10	ALL	Bad APE boots and high battery temperatures
2019/141	5	TIM	TIM temperature is out of limit
2019/150	10	TIM	TIM temperature is out of limit

### Reaction Wheel and Star Tracker Anomalies

In September 2008, reaction wheel #3 (RW-3) started showing signs of increased friction and variable performance. Analysis indicated that including the under-performing wheel in the attitude control system was impacting pointing stability. After several weeks of analyzing performance, it was decided to turn this wheel off. SORCE had four reaction wheels, with one being a redundant wheel. Flying using three wheels had almost identical pointing quality to flying with all four wheels; consequently, the three-wheel operations had little impact on science return. In December 2016, there were several orbits where torque commands were sent to RW-3; however, there was no indication that the wheel moved in response to these commands, so this wheel was treated as unusable for the remainder of the mission. The remaining three reaction wheels operated nominally with no signs of increased friction for the full mission duration of 17 years.

In September 2012, star tracker #2 failed. The failure was not the result of a gradual degradation but rather an instantaneous failure within the sensor electronics. The observatory was launched with two star trackers, and star tracker #1 continued to perform nominally through the full mission. The loss of star tracker #2 had no impact on science return.

### Degraded Battery Capacity and Daylight-Only Operations

**Figure 4:**
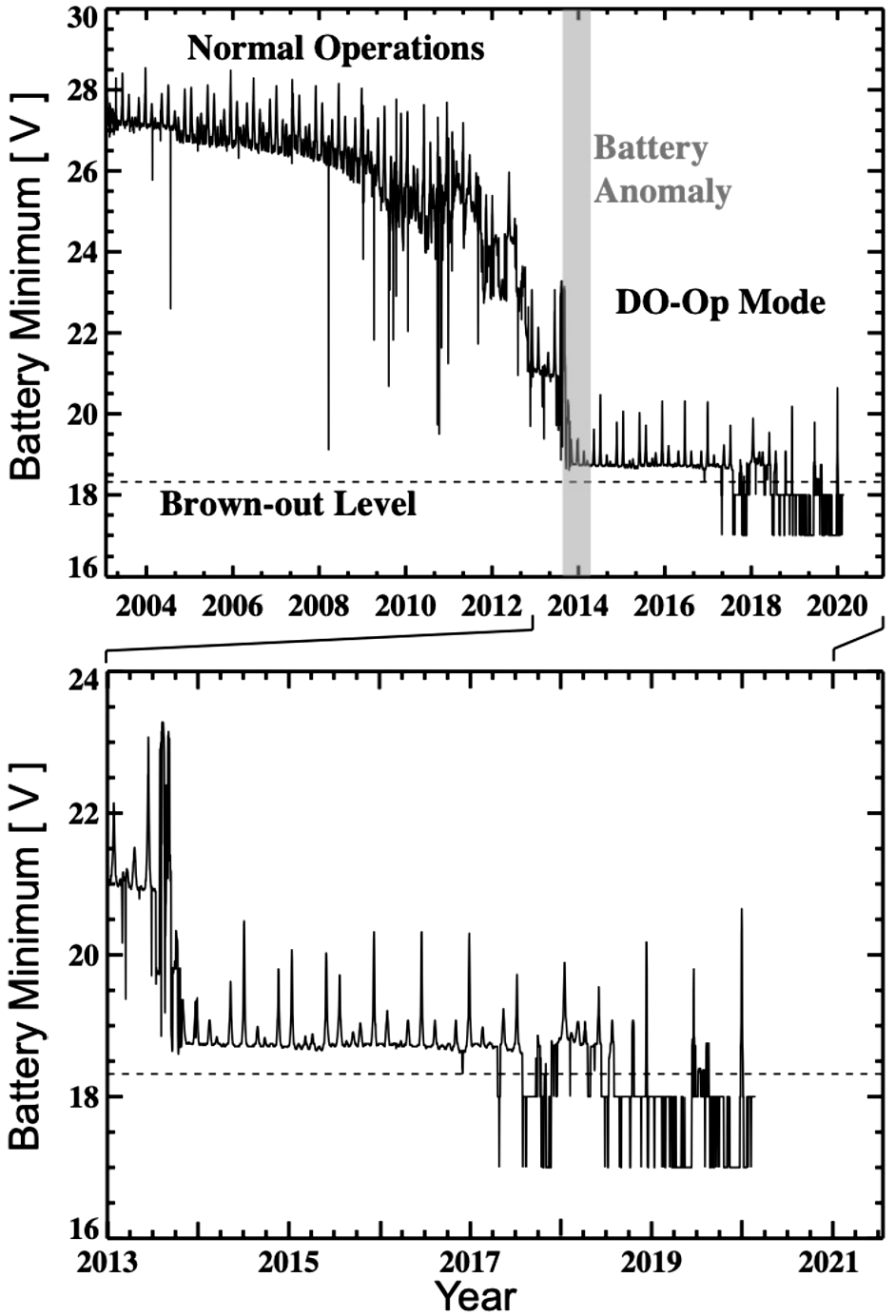
The SORCE battery voltage (capacity) slowly degraded during the first six years of the mission. Then starting in 2009, there were 1.3 V drops for each CPV cell loss. The SORCE battery became more stable once that Day Only Operations (DO-Op) mode was implemented in 2014. Mission operations were also modified for brown-out conditions whenever the battery voltage fell below 18.32 V as indicated by the dashed horizontal line. Flight software changes allowed SORCE to continue solar observations even in this brown-out mode. The voltage sudden drops and most of the downward spikes are from battery cell failures. The short-duration voltage rises are mostly due to orbit precession when the orbit eclipse period is shorter (10 minutes instead of 20 – 30 minutes) and thus the battery is discharged less.

The aging of the SORCE battery was the most troublesome component during the extended mission. As the battery capacity degraded, as illustrated in Figure 4, the SORCE Flight Operations Team (FOT) tailored the mission operations to match the loads powered on during orbit eclipses to the capacity of the battery. The battery is composed of 11 Nickel–Hydrogen Common Pressure Vessels (CPVs). Each CPV contains two battery cells. During a deep discharge event in 2009, the FOT identified six CPVs with abnormal cell behavior. Over the next several years, each of these suspect CPVs started to under-perform. As battery cells failed, non-critical subsystems were turned off in orbit eclipse, and all systems turned on during orbital daylight so that SORCE could continue daily solar observations. In July 2013, the battery performance degraded to the point where the spacecraft On-Board Computer (OBC) also needed to be power cycled around orbit eclipse. In response, the FOT developed a new operation mode that is defined here as the Daylight-Only Operations (DO-Op) mode. This required significant updates to the flight software and consequently resulted in a seven-month SORCE data gap, the longest of the mission. Routine solar observations were restarted in March 2014 and continued until the last day of the mission, when SORCE was purposedly passivated.

The concept of operations in DO-Op remained unchanged since the original implementation. In orbit eclipse, the observatory spins at ≈0.5° per second and is generally pointed within 45° of the Sun vector with only the Attitude Power Electronics (APE) and a few essential attitude determination components turned on. When the coarse Sun sensors detect that the spacecraft has exited eclipse, it declares sunrise, powers on the reaction wheels and magnetic torque bars, points towards the Sun, and zeros out spacecraft body rates by transferring momentum from the spacecraft into reaction wheels. Once the solar-array current has been above 20 amps for more than 60 seconds, the APE turns on the OBC and instrument Microprocessor Unit (MU), which would power on and configure the instruments into a default configuration. As the OBC boots, it executes a stored command sequence to turn on the remaining components. Additionally, the OBC runs through a series of checks to validate the observatory state of health. This boot-up sequence is able to detect and correct a number of spacecraft-configuration problems. If a serious problem is detected, the observatory will remain in safe-hold mode for that orbit day. If no problems are detected, the system autonomously commands from safe-hold mode to contingency mode. As this happens, control of the spacecraft is passed from the APE to the OBC. This initialization process usually completes within about seven minutes of sunrise.

With the OBC back in control, the observatory is prepared to resume science operations. A TDRS contact is scheduled close to sunrise and a stored command sequence is loaded. The sequence contains a series of slews to keep the spacecraft pointed at the Sun throughout orbit day. Unfortunately, while in contingency mode, the spacecraft roll axis about the Sun vector is unconstrained. As a result, there are many roll angles where the Earth occults the single operational star tracker while trying to achieve normal science Sun-pointing mode. When this happens, the system simply waits seven minutes and tries again, up until approximately 20 minutes prior to eclipse entry. The on-board systems are configured to detect when the slew to science attitude has completed successfully. Once this condition is met, the instruments are commanded into science mode and begin executing solar-observation sequences that are stored in the flight computer EEPROM.

During the second half of each orbit day, additional TDRS Space Network (SN) or Ground Network (GN) contacts are scheduled to capture the data. During the TDRS contacts, the science and housekeeping data are downlinked and delivered to the MOC in real-time. During a GN contact, all of the data recorded for that orbit are downlinked. If no such contact is available, all data from that orbit, which were not captured real-time, are lost upon eclipse entry when the OBC is powered off.

Five minutes prior to sunset, the stored command sequence halts science-data collection and commands the spacecraft back to safe-hold mode. Additional instrument saving and health-preservation activities are part of the shutdown sequence. The safe-hold computer monitors for the solar-array current to fall below 20 amps and commands the non-essential loads to power off.

This sequence of turning on at sunrise, loading a stored command sequence to execute during orbit day, and turning off again at sunset occurs fifteen times per day, once for each orbit. The system in this DO-Op mode was very reliable with the spacecraft making the transition to science mode for about 89% of all orbits. This success rate far exceeds the mission requirement for four observations per day. Furthermore, the operations for the DO-Op mode were highly automated, so the Flight Operations Team (FOT) personnel did not have to work 24–7 shifts after the recovery in 2014.

The battery performance became much more stable in DO-Op mode. Increasing the battery-charging amount in mid-2013 may have helped to decrease the rate of battery degradation. Beginning in August 2016, CPV #9 started showing unique anomalous behavior. Over several months, the dayside voltage gradually increased from approximately 2.3 V to over 7.0 V. No additional capacity was gained during eclipse, however, and on 30 November 2016, there was an anomaly in CPV #9 that lowered the battery voltage while in eclipse to be below 18.34 V. This low voltage results in a brown-out (power off) of the APE. When the APE browns out, the FOT loses visibility into the minimum voltage each orbit. A second brown-out threshold can be detected when the Uplink card also browns out. When this lower threshold is crossed, all spacecraft components are powered off. The SORCE spacecraft experienced 7,225 APE brown-out events and an additional 1,967 Uplink card brown-outs. Even in these brown-out modes, the SORCE spacecraft was able to recover nearly autonomously and make routine solar observations.

By design, brown-out mode is very similar to the DO-Op mode. The following describes a brown-out orbit. At sunrise, sunlight on the solar arrays powers up the APE, and it boots to the “golden copy” of flight software that does not have any of the DO-Op modifications. In this case, recovery is initiated by ground commands to turn on the OBC during a TDRS contact early during orbit day. The flight software on the OBC then configures the APE for DO-Op mode. Next, a stored command sequence is loaded from the ground as in non-brown-out DO-Op mode. This stored command sequence would perform maneuvers for solar pointing and start science operations once normal solar science pointing was achieved.

This DO-Op mode of operations was a mission-saving success because it allowed SORCE to continue daily solar measurements and recover from brown-outs. The unique part of the SORCE instrument operations and how they evolved over the mission are described in the companion instrument articles in this *Solar Physics* Topical Collection. There are some common instrumental effects as related to the spacecraft anomalies. For example, the orbital temperature variations became larger for the instruments when power cycling began for orbit eclipses. While temperature corrections for the sensor gains and other parameters are made in the science data processing, these temperature variations do increase the irradiance uncertainties during the DO-Op mode.

### Instrument Anomalies

The SORCE instruments had very few anomalies over the full 17-year mission. We note those that were more significant here, as observations were changed in response to those anomalies. More information is provided in the individual instrument companion articles.

In December 2005, the XPS had an anomaly with its filter-wheel mechanism not moving. While it did begin to work again after this initial anomaly, we changed the operations for XPS to use the filter-wheel mechanism less frequently. Namely, the calibration experiments with redundant XPS channels were conservatively changed from daily to monthly calibrations to reduce the use of its filter mechanism.

In January 2006, the SOLSTICE-A mechanism for switching between solar and stellar apertures did not properly operate for a short period of time. Although it began working again, the entrance aperture of SOLSTICE-A was intentionally left in solar mode rather than risk further anomalous behavior. There has been no impact on SOLSTICE’s core science because the SOLSTICE-B channel continued to make both solar and stellar observations and because weekly cross-calibrations between the two SOLSTICE channels provided degradation corrections for SOLSTICE-A.

The spacecraft battery issues significantly reduced the number of stellar observations acquired by the SOLSTICE when power cycling of that instrument started in 2010. These stellar observations are a key technique for tracking instrument degradation for the SOLSTICE channels. There were just a few stellar observations in DO-Op mode, performed by off-pointing the spacecraft during orbit day. More importantly, a modified version of the cruciform field-of-view scans was developed to accurately track differences between solar and stellar exposure effects on the SOLSTICE optics. To address the long-term stability concern with very limited stellar observations, a new instrument, the Compact SOLSTICE (CSOL), was developed in 2017 and then successfully flown as a rocket calibration underflight in June 2018. The CSOL calibrations and results are discussed by Thiemann et al. (2021).

## Summary

The SORCE/TSI and SORCE/SSI observations are key climate-monitoring measurements and were made with unprecedented accuracy and measurement precision. Three important accomplishments of the SORCE mission are i) the continuation of the 42-year-long TSI climate record, ii) the continuation of the ultraviolet SSI record, and iii) the initiation of the near-ultraviolet, visible, and near-infrared SSI records. The SORCE instruments and spacecraft functioned very well over the 17-year mission, which far exceeded its 5-year prime mission goal. More specifically, the SORCE mission made solar observations from 25 February 2003 to 25 February 2020 and provided daily averaged solar irradiances for 95% of the days. There are 17 periods, totaling 296 days, during the 17-year mission (6209 days) without any solar observations, with the majority (210 days) being caused by a significant battery-capacity issue causing data gaps between August 2013 and February 2014. The end of the SORCE mission was a planned passivation of the spacecraft following a successful two-year overlap with the TSIS-1 mission, which continues the TSI and SSI climate records. The TSIS-1 SSI observations span 200 to 2700 nm, extending further into the IR than SORCE but leaving a spectral gap below 200 nm. The new NOAA GOES-R series of satellites provide limited SSI coverage (0.1 – 0.8 nm, 25 – 31 nm, 117 – 141 nm, and 279 – 281 nm) to fill part of this spectral gap (Machol et al., 2020). Companion articles about the SORCE instruments and their final science data-processing algorithms provide additional details about individual instrument measurements over the course of the SORCE mission.

## Data Availability

The SORCE public data products are described on the SORCE website at lasp.colorado.edu/sorce/data/. Data files, and software to read them, are available for direct download from this web site. SORCE data products are delivered and archived at the Goddard Earth Science (GES) Data and Information Services Center (DISC) at disc.sci.gsfc.nasa.gov/SORCE/data-holdings. In addition, the LISIRD website at lasp.colorado.edu/lisird/ provides interactive access to the SORCE solar-irradiance data, where they coexist with related solar-irradiance data products from other missions.
